# Perspectives of professionals on the perinatal period as a window of opportunity for change in women with SUD: insights from healthcare, child welfare, substance use, and child protection services

**DOI:** 10.3389/fpsyt.2026.1668040

**Published:** 2026-02-04

**Authors:** Sarah Vandewalle, Sara Rowaert, Gilbert Lemmens, Sarah De Pauw, Wouter Vanderplasschen

**Affiliations:** 1 Department of Special Needs Education, Faculty of Psychology and Educational Sciences, Ghent University, Ghent, Belgium; 2 Department of Head and Skin, Faculty of Medicine and Health Sciences, Ghent University, Ghent, Belgium; 3 Department of Psychiatry, Ghent University Hospital, Ghent, Belgium

**Keywords:** perinatal period, professionals’ perspectives, strengths-based, substance use, window of opportunity

## Abstract

**Introduction:**

The perinatal period is widely recognized as a time of profound transition and heightened vulnerability, particularly for women with substance use disorders (SUD). Adopting a strengths-based perspective, this qualitative study aims to explore how professionals across healthcare, child welfare, substance use, and child protection services in Belgium perceive this period as a window of opportunity for change in women with SUD.

**Method:**

Semi-structured interviews were conducted with 43 professionals, and thematic analysis identified key themes related to barriers, opportunities, and facilitators to achieve change.

**Results:**

Barriers include disconnected bodily awareness, mutual avoidance between women and professionals, limited insight and (self-) reflection on SUD and motherhood, and insufficient support across interpersonal, socioeconomic, and systemic levels. However, professionals also identified several areas that could be reinforced to unlock the period’s transformative potential, reflecting a gradual shift from a deficit-oriented view toward a more strengths-based understanding of perinatal SUD. Empowerment was seen as crucial through open conversations on family planning, psychoeducation on fertility and contraception, access to appropriate contraceptives, and trauma-sensitive, body-oriented approaches that foster connection to pregnancy and the unborn child, as well as the integration of the infant mental health perspective. The perinatal period itself was considered a naturally occurring window of opportunity for change, driven by processes of identity transformation and growing maternal motivation. Professionals emphasized the importance of enhancing professional capacity, particularly in healthcare, and improving screening and referral by midwives and gynecologists. At the care system level, increased and more consistent contact with healthcare services during pregnancy was described as a contextual opportunity for timely support; however, intersectoral collaboration and integrated care were considered essential, alongside a legal prenatal framework that enables early, non-punitive interventions to support both mother and (unborn) child.

**Discussion:**

These findings underscore the need to move beyond hegemonic discourses that frame strength and deficit, mothering and substance use, or vulnerability and opportunity as binary opposites. Recognizing the ways these dimensions coexist and intersect is vital for developing responsive, relational, and ethically grounded models of perinatal care.

## 1 Introduction

Pregnancy up to one year after delivery (“perinatal period”) is a highly challenging period for most women and is now increasingly recognized as a time of heightened vulnerability (1–4). Women need to deal with profound and rapid physiological transitions, to adopt new identities, and adapt to changing family and social roles (5). This is in particular the case for women with substance use disorders (SUD) (6, 7).

Recently, perinatal SUD has emerged as a growing global public health concern (8–11), as women of childbearing age are increasingly affected by SUD (10, 12, 13) and the postpartum period is recognized as one of the biggest times of risk for the use of any substance (14). Estimates of perinatal SUD vary widely and remain difficult to establish due to methodological challenges and underreporting (6, 15). In the United States, recent data from the National Survey on Drug Use and Health indicate that past-month illicit substance use among pregnant women ranges from 4.67 to 14.81% (16). In Europe, approximately 4.5% of all pregnant women use illicit substances, and between 6.5% and 11% of women with SUD become pregnant or give birth each year (8). In Australia, a recent population-based study reported that 3.4% of all children had a record of maternal SUD during the first 1000 days of life, including 1.4% with documented prenatal substance use (17). A recent Belgian study based on self-reported questionnaires found that 14.6% of women consumed alcohol during pregnancy, and 0.4% used illicit drugs. These rates are approximately ten times lower than those reported in similar studies from other countries, likely due to underreporting and social desirability bias (18).

Pregnant and parenting women with SUD are often exposed to several, multilayered forms of stigma, posing significant barriers to (mental health) care. Societal stigma (such as “pregnant women struggling with SUD are bad mothers/persons”, “are weak”) often intensifies during pregnancy (19–21), and societal expectations around motherhood—such as the ideal of the “good mother”—often clash with the lived realities of women with SUD, reinforcing shame and internalized stigma (8, 20). Also, structural and enacted stigma within healthcare settings (such as punitive treatments, mistreatment, and judgment) can reinforce professionals’ prejudices towards these women and increase barriers to receiving care (22–24). Finally, anticipated stigma (e.g., fear of involvement from child welfare services and loss of custody) can deter women from disclosing substance use, symptoms, or even the pregnancy itself, and from seeking help (24, 25). Indeed, infants prenatally exposed to substances are 11 times more likely to be placed in foster care (26), and approximately half of all mothers in substance use treatment have lost custody of at least one child (27–29). Consequently, pregnant and parenting women with SUD remain underrepresented in substance use treatment (30–33) and often avoid or delay prenatal care, increasing the risk of both maternal and infant morbidity and mortality (34, 35). In the United States, the postpartum period is increasingly recognized as a time of vulnerability, with overdose now recognized as the second leading cause of maternal death (32, 34, 35). Beyond substance-related risks, women with SUD are also at heightened risk for postpartum depression (7). Moreover, the compounding effects of multiple stigmas, stress, and limited access to care often result in relapse within the first year after childbirth (6, 7).

Recently, strength-based research and approaches have begun to challenge these widespread stigmas, as well as punitive and deficit-oriented responses to perinatal SUD (36, 37). Since pregnancy and childbirth can serve as powerful turning points, prompting self-reflection, identity changes, and a re-orientation of goals and values (22, 38–40), pregnant and parenting women with SUD are often highly motivated to initiate behavioral changes (14, 41, 42) and to increase engagement with services. Indeed, many initiate treatment for SUD during the perinatal period, either on their own initiative or after ‘encouragement’ from care providers (8, 42, 43). Furthermore, the maternal concern for the child’s development and well-being (44), alongside fear of custody loss, can serve as an additional strong motivator for change (3, 41) and seeking treatment. In this context, the (unborn) child is increasingly understood as an active agent in the recovery process (43, 45). Finally, increased contact with healthcare services during the perinatal period (42, 46) offers many opportunities to engage with these women and their families (47), to deliver risk-responsive prenatal care (46), to implement therapeutic interventions (3), and to capitalize on women’s enhanced motivation (36), while potentially mitigating the risk of mother-infant separation (48).

To date, research on the perinatal period as a ‘window of opportunity’ has predominantly focused on women with SUDs’ perspectives (38, 40, 43, 46) or on the effectiveness of specific interventions and treatment programs (3, 41, 45, 47). In contrast, the perspectives of professionals who come into contact with these women during the perinatal period remain relatively underexplored, with existing studies primarily focusing on medical professionals, such as gynecologists and midwives (42, 48, 49), while limited attention has been given to the insights of professionals in substance use services, child welfare, and child protection services (22). Moreover, existing research has adopted a rather deficit-oriented perspective, emphasizing the “burden of care” or the challenges and difficulties professionals face in managing perinatal SUD (19, 49, 50).

Consequently, the current study aims to adopt a more strengths-based focus, exploring the perspectives of professionals working in four different domains: healthcare, child welfare, substance use, and child protection services. In Belgium, substance use and healthcare services are primarily funded through the national, mandatory health insurance system, which reimburses treatment and medical care costs and is available to all citizens. Specific perinatal projects have been initiated at the local level, targeting pregnant and parenting women who use substances, aimed at accessing and monitoring this hard-to-reach population and coordinating substance use and medical services. Child welfare and child protection services, financed at the regional level, often become involved in cases of parental substance use.

These four sectors were selected because of their central role in the Belgian perinatal care landscape, where support for pregnant and parenting women with SUD is typically coordinated across these domains. In Flanders, for example, professionals from obstetric and pediatric care, child welfare, substance use services, and child protection agencies are often jointly involved in assessing risk, providing support, and making decisions about family preservation or child placement. However, the mandate of child welfare and child protection services to prevent child abuse and promote children’s well-being can sometimes conflict with the supportive approach of substance use services toward pregnant and parenting women, making coordinated care and case management that takes into account children’s and parents’ interests essential, yet its availability is limited and varies by region.

Understanding how professionals across these sectors perceive the perinatal period is therefore essential to improving cross-sectoral collaboration and developing more responsive, integrated care pathways. The central research questions are:

1. How do professionals working with pregnant and parenting women with SUD perceive the perinatal period as a ‘window of opportunity’ for change?
2. What can be done to enhance the transformative potential of the perinatal period for these women?

## 2 Methodology

### 2.1 Research design

This study explores the perspectives of professionals working at the intersection of perinatal care, parenting, and SUD. A qualitative research approach was employed, using semi-structured interviews to enable an in-depth examination of participants’ views while ensuring consistency in addressing key topics across interviews.

The paper was reported according to the Consolidated Criteria for Reporting Qualitative Research (COREQ) checklist for qualitative studies (51).

### 2.2 Participants

Participants were recruited through a combination of purposive and snowball sampling. Initial recruitment involved contacting all relevant organizations across Flanders, Belgium, that offer specialized services for pregnant and parenting women, facing SUD (see *, **Table 1**). These organizations included residential substance use services, as well as outpatient (and outreach) initiatives offering family support, intensive case management, and assistance in building supportive caregiving networks. Subsequently, snowball sampling was employed, whereby initial participants and organizations were asked to refer additional relevant contacts with expertise in the topic ‘perinatal substance use’, thus expanding the participant pool. For the purpose of this study, “SUD” was not operationalized through direct diagnostic assessment but reflected the terminology used by professionals during the interviews and in clinical practice. Professionals typically referred, in the interviews, to women they worked with — pregnant or parenting women — who had a SUD and engaged in active substance use during the perinatal period.

**Table 1:** Overview of participants.

Interview (participant)	Sector	Type of organization	Job function	Years of professional experience
1	HC-1	General hospital	Gynecologist	>10
2	SU-1	Detoxification program (pregnant) parent + child*	Coordinator	>10
3	SU-2	Outpatient support for (pregnant) parents with SUD*	Case-manager	>10
4	SU-3	Outpatient support for (pregnant) parents with SUD + perinatal support network for SUD*	Psychosocial worker – coordinator	>10
5	CPS-1	Child protection and family assessment agency	Team coordinator	>10
6	CW-1	Center for childcare and family support	Family support worker	>10
7	SU-4	Outpatient support for (pregnant) parents with SUD + Perinatal support network for SUD*	Coordinator	>10
8	CPS-2	Early intervention for high-risk infants	Case coordinator	5-10
9	SU-5	Outpatient SUD services + perinatal support network for SUD*	Psychosocial worker	>10
10	HC-2	Mental health care network	Early childhood specialist	0-5
11	SU-6	Outpatient support for (pregnant) parents with SUD*	Family support worker	5-10
12	CW-2	Residential family care institution	Director	>10
13	HC-3	General hospital	Psychiatrist	>10
14(A)	SU-7	Program within Therapeutic Community, specific for (pregnant) parents*	Family support worker	0-5
14(B)	SU-8	Program within Therapeutic Community, specific for (pregnant) parents*	Family support worker	>10
15	HC-4	University hospital	Pediatrician	>10
16	CW-3	Residential family care institution	Case coordinator – family support worker	5-10
17	SU-9	Outpatient substance use services	Psychosocial worker	>10
18(A)	HC-5	General hospital	Head of midwifery	>10
18(B)	HC-6	General hospital	Social Worker	>10
19	CPS-3	Child abuse trust center	Physician specialized in child protection concerns	>10
20	CW-4	Center for childcare and family support	Family support worker	0-5
21(A)	CW-5	Child & family services	Social worker – family support worker	>10
21(B)	CW-6	Child & family services	Social worker- Family support worker	>10
22	SU-10	Outpatient support for (pregnant) parents with SUD*	Coordinator	>10
23(A)	HC-7	General hospital	Gynecologist	5-10
23(B)	HC-8	General hospital	Social worker	0-5
24	SU-11	Outpatient substance use services + perinatal support network for SUD*	Psychosocial worker - Coordinator	>10
25	HC-9	Integrated perinatal care program for vulnerable mothers + University hospital*	Perinatal Coach + midwife	>10
26	CW-7	Residential child welfare	Coordinator	>10
27	CW-8	Foster care	Family support worker	5-10
28	HC-10	University hospital	Gynecologist	>10
29	CW-9	Residential child welfare	Coordinator	5-10
30	CW-10	Outpatient family & child welfare	Family support worker	5-10
31	SU-12	SUD treatment unit, psychiatric hospital	Psychologist – policymaker	>10
32	HC-11	Mental health care network	Coordinator - policymaker	>10
33	CPS-4	Child abuse trust center	Criminologist	0-5
34	CW-11	Child & family services	Social worker- Family support worker	5-10
35(A)	CPS-5	Perinatal support project within child protection and family assessment agency*	Youth Protection Officer	>10
35(B)	CPS-6	Perinatal support project within child protection and family assessment agency*	Youth Protection Officer	0-5
36	CPS-7	Child abuse trust center	Psychologist	>10
37	HC-12	General hospital	Pediatrician, policy maker	>10
38	CW-12	Center for childcare and family support	Coordinator	>10

* services specialized in perinatal SUD.

After providing written informed consent, a total of 43 professionals participated in 38 interviews, including 10 paired interviews, resulting in more participants than interviews (see **Table 1**). As part of the consent procedure, participants were informed that the study was embedded in a research project exploring whether and how the perinatal period may provide opportunities for change and recovery for women with SUD. Participants were drawn from four sectors:

- Substance use services (SU, n=12): These professionals provide treatment, support, and outreach for individuals with SUD. Some services specifically target pregnant and parenting women with SUD, or parents in general, offering tailored support.
- Healthcare (HC, n=12): This group includes professionals involved in perinatal medical care, such as obstetricians, pediatricians, midwives, and social workers
- Child welfare (CW, n=12): These services offer preventive, supportive, and—when necessary—mandated care to children and families. They aim to strengthen family functioning and promote child well-being through both residential and outpatient support.
- Child protection services (CPS, n=7): These agencies are responsible for evaluating concerns about child welfare and determining whether intervention is needed to ensure the child’s safety and development. They assess whether protection measures are warranted, coordinate and monitor support services, and, when necessary, refer cases to the juvenile court for formal judicial protection.

No participants dropped out during the study.

### 2.3 Data collection

Data collection took place between February and April 2024. All interviews were conducted by the first author (SV). The interviews lasted between 23 minutes and 97 minutes. Depending on the participant’s preference, interviews were conducted in person (n=25), online (n=12), or by telephone (n=1). All interviews were audio-recorded. No field notes were made during the interviews.

A semi-structured interview guide was used, consisting of standardized open-ended questions designed to ensure consistency across interviews while allowing for in-depth exploration of professionals’ individual experiences and perspectives. The interview guide is available upon request from the first author (SV).

Interviews started with general questions regarding how professionals encounter pregnant or parenting women with SUD in their work, and how these issues are identified and addressed within their specific organizational context. Participants were also asked to describe their approaches to care and support, including engagement strategies and collaboration with other services. The conversation then shifted to the intersection of parenting, child welfare, and SUD. Professionals were invited to reflect on the factors that influence whether a mother and child could remain together, and to share their experiences with custody loss and decisions regarding out-of-home placement. These discussions aimed to elicit the values, considerations, and structural factors (such as policy regulations, the availability and accessibility of services, and legal frameworks) that shape professional decision-making.

Specific attention was given to exploring the potential of the perinatal period as a ‘window of opportunity’. To this end, participants were explicitly invited to reflect on the following question: “*The perinatal period is often described as a ‘window of opportunity’* (41, 42, 45) *for women with a substance use problem, partly due to increased motivation for change and recovery. How do you perceive this idea, and how does this resonate with your experiences in practice?”* This line of questioning examined whether professionals observed heightened motivation, openness to change, or increased engagement during this period, and under what conditions such opportunities were perceived to arise or could be supported.

Finally, participants were asked to share their views on how support for pregnant and parenting women with SUD could be improved. Within this discussion, attention was paid to strengths-based approaches: professionals were asked to identify protective or facilitating factors in women’s lives that promote change. These questions allowed participants to articulate both individual and structural facilitators as well as barriers to change, along with their implicit beliefs about recovery potential and maternal competencies during the perinatal period.

Data collection continued until saturation was reached, meaning that sufficient and redundant information had been obtained and no new insights emerged from additional interviews.

### 2.4 Data analysis

All interviews were transcribed verbatim. Participants were given the opportunity to review and, if desired, amend their interview transcripts prior to the commencement of analysis. Only one participant opted to review the transcript, but no corrections or comments were made. Data was analyzed using Braun and Clarke’s 6-phase thematic analysis approach (52). The primary analysis was conducted by the first author (SV). The process began with a thorough familiarization with the data through repeated listening to the recorded interviews and careful reading of transcripts. During this phase, initial codes were generated inductively, capturing meaningful features of the data relevant to the research aims. These first-round codes were very broad initially, for example, “need for psychoeducation”, “knowledge about the impact of substance use”, or “access to contraception”, and served to summarize recurring content across interviews.

In the subsequent step, these initial codes were reviewed, grouped, and color-coded to facilitate organization and clustering. This coding process enabled the emergence of overarching themes and subthemes related to barriers, opportunities, and facilitators to achieve change during the perinatal period. Particular attention was given to ensuring subthemes reflected perspectives from professionals across the key sectors - healthcare, substance use services, child welfare, and child protection services – grounding the findings in the cross-sectoral nature of the sample rather than reflecting a single welfare domain. This cross-sectoral focus arose naturally, as the themes consistently emerged across all four professional sectors. While sector-specific perspectives added nuances, the overarching dynamics of change in the perinatal period were shared broadly, reflecting the multidisciplinary nature of the sample.

Sector-specific nuances included, for instance, the importance of the Infant Mental Health (IMH) perspective, which was primarily articulated by professionals working in child welfare, while professionals in substance use services highlighted the role of early trauma and dissociation in cases of pregnancy denial. These sector-specific insights were integrated into broader cross-sectoral themes as illustrative nuances. This approach allowed for both thematic coherence and representation of sectoral depth.

Themes were iteratively reviewed and reorganized to ensure coherence, internal consistency, and representativeness of the data. All transcripts were re-read to confirm that the developed themes adequately captured the breadth and depth of the data. Throughout the process, the first author (SV) discussed emerging codes and themes with the second author (SR). Themes were further refined through an iterative process in collaboration with all co-authors (SV, SR, GL, SDP, WV), experienced in conducting qualitative research, strengthening the interpretive coherence of the findings. Quotations used to illustrate the themes were translated into English by the first author (SV), with efforts made to remain as close as possible to the original language while ensuring clarity and readability.

The two main themes identified were (1) “Barriers to engagement and change” and (2) “Opportunities and facilitators to achieve change”, as perceived by professionals. Each theme encompassed four subthemes. **Table 2** provides an overview of these themes and subthemes.

**Table 2:** Overview of themes and subthemes.

Main theme	Subthemes
Barriers to engagement and change according to professionals	- Disconnected bodily awareness
	- Mutual avoidance in care relationships
	- Limited insight & (self-)reflection on SUD and motherhood
	- Insufficient support networks and systemic gaps
Opportunities and facilitators to achieve change according to professionals	- Supporting reproductive empowerment and embodied connection
	- Motherhood as a motivational and identity shift	- Enhancing professional capacity and relational practice
	- Building an integrated and responsive care system

### 2.5 Ethical considerations

This research was conducted in accordance with the ethical regulations outlined in the General Ethical Protocol of the Faculty of Psychology and Educational Sciences at Ghent University, which is based on the European Code of Conduct for Research Integrity (53). Prior to participation, both oral and written informed consent were obtained from all participants, using a consent template developed by the Ethical Committee of the Faculty of Psychology and Educational Sciences. Participants were informed about the study’s purpose, their right to withdraw at any time, and the confidentiality measures implemented to protect their identities and data.

## 3 Results

Interview findings show that professionals across health, child welfare, substance use, and child protection services generally acknowledged the potential of the perinatal period to facilitate change for pregnant and parenting women with SUD. However, their accounts were often followed and nuanced by significant remarks, reflecting varying levels of confidence in its transformative power and highlighting the complex, often ambivalent nature of this period, specifically in cases of perinatal SUD.

First, we discuss the barriers that highlight the personal, contextual, and systemic challenges women with SUD and professionals must navigate to activate strengths and opportunities for change, such as disconnected bodily awareness and mutual avoidance in care.

Second, we discuss opportunities and facilitators to achieve change during this period according to professionals, e.g., the transformative role of motherhood as a motivational and identity shift, and strategies to support reproductive empowerment and embodied connection.

### 3.1 Barriers to engagement and change according to professionals

Professionals across sectors identified a range of barriers that can hinder change during the perinatal period, including disconnected bodily awareness; mutual avoidance in care relationships; limited insights & (self-)reflection into substance use, parenting, and motherhood; and insufficient support at (inter)personal, socioeconomic, and institutional levels.

#### 3.1.1 Disconnected bodily awareness

Several professionals stated that women believed they were infertile due to the absence of menstruation related to SUD, malnutrition, or poor living conditions. As one professional explained:

*“Sometimes they are more fertile than they think they are … Actually, a lot of them think they are not fertile”* (SU-4).

This misunderstanding frequently led to incorrect or absent contraceptive use, increasing the likelihood of unprotected sex and unintended pregnancies.

Some professionals reported that many of these women often remained unaware of physical changes associated with pregnancy until late in pregnancy, particularly among women with long-standing SUD and trauma histories. This often resulted from their misconceptions about fertility, and the women not feeling connected with bodily sensations.

*“Another woman who gets pregnant knows after a few weeks that something is changing in her body. These women only find out at month seven that they are pregnant.”* (SU-11)

*“She called me two days after that, [ … ] she was 36 weeks pregnant … She hadn’t noticed.”* (CW-12)

Professionals mentioned that the late or unexpected pregnancy discovery could lead to (more) substance use. First, the stress associated with the sudden realization of pregnancy could lead to increased substance use:

*“We sometimes hear from women that they initially used a lot more when they knew they were pregnant [ … ] That, for them, in fact, it just led to more use rather than less use…”* (SU-1)

Second, when pregnancies remained unrecognized, substance use typically continued through critical stages of fetal development. Professionals across all sectors illustrated that the awareness of the harm already done often triggered feelings of guilt and anxiety, which could in turn lead to the continuation of drug use or even relapse after stopping substance use later in pregnancy (“no point of stopping since harm was already done”):

*“Eventually, at a certain point, you realize that you’re pregnant, but by then, a very critical period of the pregnancy’s development has already passed. There is already scientific evidence showing that changes may have occurred in the baby’s brain that will last a lifetime, even if you stop [using]. So, I believe that sense of guilt is inevitable as well.”* (HC-4)

#### 3.1.2 Mutual avoidance in care relationships

Although professionals emphasized the importance of early intervention, mutual avoidance between pregnant women and professionals frequently resulted in late engagement with care services, sometimes only after childbirth.

Professionals observed that some women managed to hide their pregnancy or substance use for a considerable time. This intentional concealment was often motivated by fear of stigma and punitive consequences, particularly the involvement of child protection services and potential loss of custody:

*“She would go with the child protection officer [ … ] and then she would wear extra loose clothes so that the social worker wouldn’t know she was pregnant, and she actually managed to keep that up almost until the end of the pregnancy.”* (SU-11)

This concealment often went unnoticed, even until admission to the maternity ward:

*“People hold on very strongly until they finally get caught, and that can sometimes be late in the pregnancy, sometimes even in maternity ward, yeah, women where we haven’t noticed much during the whole pregnancy, because they only have to hold on for a quarter of an hour per month to see a doctor, and then in maternity ward, yeah, they break down, because then we see them 24 hours.”* (HC-9)

Moreover, professionals stated that some women actively avoid contacting healthcare services to remain undetected, leading to serious discontinuities in care:

*“For example, these could be people from Brussels or the other side of Belgium, who just go give birth somewhere in order to stay under the radar”* (HC-5).

Professionals across all four sectors also highlighted shortcomings within the healthcare system that contribute to the underdetection and referral of substance use during pregnancy.

First, routine screening is seldom systematically implemented. Several professionals attributed this to a lack of confidence or competence, fear of damaging the therapeutic relationship, or the assumption that another professional will take responsibility:

*“What we notice, for example, is that we often receive reports about mothers, mostly from the same hospitals, but almost never from others. That can’t be right; it says something about … the vigilance you want to have, or … awkwardness, because we think ‘someone else will pick it up’.”* (CPS-3)

Others expressed feeling ill-equipped to address prenatal SUD due to limited knowledge about its effects and a lack of clarity on referral pathways. Professionals referred to discomfort in initiating the conversation, combined with uncertainty about what actions might follow:

*“A bit of reluctance to question it, because it’s a threshold, but also ‘What about … what about the information we get then?’”* (SU-5).

Moreover, concerns about jeopardizing trust were described as barriers, as professionals feared that raising the issue could cause women to disengage from care:

*“Report or not? Yeah. And will we lose them then?”* (CPS-7).

Second, when screening does take place, its quality is often inadequate. Professionals indicated that the way screening is conducted can vary considerably, even when protocols are available. They emphasized that the phrasing of questions may discourage honest disclosure, and that screening is sometimes only applied to women who fit a particular risk profile, reflecting appearance-based assumptions and biases:

*“I can imagine that here and there it’s sometimes asked in a very subjective way like, ‘You’re not using anything, right?’”* (HC-9)

*“And I find that a bit strange, that it’s just assumed, if you’re at a normal age, if your partner comes along … if you’re white, if you … That there’s just … Yeah, I find it striking that that’s just assumed like that.”* (CW-8).

As a result, women who do not match these expected risk profiles may go undetected, and opportunities for early support are missed.

Third, professionals noted that attitudes toward substance use during pregnancy vary significantly among healthcare providers. While some take a very strict stance, others are overly permissive or dismiss the potential harm of substances like cannabis or alcohol during pregnancy. This ambivalent attitude may contribute to inequitable care and missed referral opportunities:

*“Some are very strict … Uh, we have a pediatrician here in the region who, the moment he hears there’s drug use, basically doesn’t want to let the child go home with the mother. That’s a bit, that’s a bit short-sighted. [ … ] And in the other direction as well, yeah, gynecologists who might then, perhaps, be too lenient, or pediatricians who might go too…, too lenient.”* (SU-9)

*“Well, personally, I’m not really that opposed to it, but of course, as a doctor during a pregnancy, yeah, you obviously can’t go around promoting cannabis use…”* (HC-1)

#### 3.1.3 Limited insight & (self-)reflection on SUD and motherhood

Professionals frequently described limited insight and self-reflection of the women on the impact of substance use on the (unborn) baby and an idealized perspective on motherhood during the perinatal period as a key barrier in initiating change.

First, professionals illustrated that many women often do not consider their substance use problematic.

*“Most of the time, they simply don’t see that there is a problem … They’ve been used to living in certain circumstances their whole life.”* (HC-1)

Secondly, professionals observed that there is little awareness about the toxic effects of substances during pregnancy because the fetus and the consequences of substance use are perceived as something abstract and vague. The fetus is “not really there yet“, and the consequences of using substances are equally difficult to grasp — they are often delayed, diffuse, and not immediately visible:

*“She doesn’t really get that the baby is already living inside her.”* (CW-1)

This difficulty in conceptualizing long-term consequences is reinforced by the lack of immediately visible effects, particularly if the baby appears healthy at birth. As one professional illustrated,

*“People are very focused on the ‘now.’ Like, “Well, if that doesn’t increase the risk of heart defects, then okay”* (HC-7).

The delayed and often less tangible nature of certain outcomes makes it harder to link present behaviors to future impacts:

*“Then we say, ‘Look, when your baby is born, the chance is very real that it will also go through withdrawal [ … ].’ And then those babies are born, and they don’t go through withdrawal. And then it’s hard to explain like, ‘Yeah, but wait … maybe not right away, but maybe it will be a baby … a baby with excessive crying, or you’ll see other signals, or it will be a poor feeder or … [the baby will have a poor] suck reflex’.”* (CPS-6)

Professionals described how this short-term focus also affects how women anticipate parenthood. Many hold an idealized view of motherhood, leading to unrealistic expectations and postponing change:

*“That’s also something they often say: ‘Yes, but once the baby is here, then I’ll stop using’.”* (CPS-6)

Further, the societal image of motherhood, one that romanticizes parenting and minimizes its emotional, practical, and financial demands, often misleads women. As a result, women may underestimate the difficulties or challenges that raising a child entails. Knowledge about what children need to grow up safely and emotionally supported is frequently lacking.

*“Having a family is hard work. And it’s very difficult to combine that with substance use [ … ] and that, that’s the societal image.”* (SU-11)

*“I don’t think parents realize that having a baby is an additional stressor and does not ease your life. Neither organizationally, which they are already having a hard time with, nor financially [ … ], and then the unavoidable sense of guilt.”* (HC-4)

In addition, the impact of ongoing substance use on children is often trivialized. Professionals reported hearing statements like:

*“The children don’t suffer from it, they don’t know [it], I use in the bathroom, my child doesn’t see it…” (SU-5) or “My child doesn’t notice that, no, really — they don’t notice it. No one can tell by looking at me”* (CPS-3).

This reasoning may not only reflect limited understanding but also serve as a coping strategy. By distancing themselves from the (unborn) child or downplaying the consequences, women protect themselves from overwhelming guilt.

*“Because, of course, if you know it and you acknowledge it, then action is expected from you. But if you don’t feel capable or don’t get around to doing it, well … then you feel guilty. And I think people often try to get rid of that guilt a bit, by minimizing — ‘yeah, but I didn’t drink that much’ or…”* (CW-7)

#### 3.1.4 Insufficient support networks and systemic gaps

One of the main difficulties in achieving change during the perinatal period is the pervasive lack of support across multiple levels. At the interpersonal level, many women lack a stable and supportive social network:

*“I think a network is also one of the really important things, in such a moment, when someone is completely alone, yeah, no one can raise a baby alone”* (SU-4).

The presence or absence of a supportive partner also plays a decisive role. While some partners may contribute positively to the recovery process, others are unsupportive or violent, increasing the women’s vulnerability. Even when pregnant or parenting women intend to change, a partner can pose a persistent relapse risk. Professionals stress the importance of empowering mothers to prioritize their child’s well-being, even when it means distancing themselves from harmful relationships.

*“It really depends, of course, on the context of that person too, eh, if she has a uhm partner who also uhm wants to make a positive story of it or is able to make one, or … [ … ] Versus a partner who doesn’t support her at all, who is violent and so on and so forth. That’s when it gets hard.”* (HC-10)

*“Then we hope that they have grown so much in their mothering role that they will say ‘No’ to all other risk factors, [ … ] that they will say to their partner ‘No, I have grown as a mother’.”* (CW-2)

Additionally, substance use rarely exists in isolation and is often entangled with broader socioeconomic and personal difficulties: housing instability, financial precarity, mental health problems, intellectual disabilities, or previous child protection involvement. The accumulation of risk factors, combined with the limited timeframe of the pregnancy period, makes lasting change extremely difficult.

*“In nine months, you can sometimes get a few things organized, but you can’t get their whole life path back on track … It’s disappointing what you can do.”* (HC-1).

Moreover, the vulnerability of this period concerning mental health issues was stressed, specifically in women with a history of severe traumatic (childhood) experiences:

*“Pregnancy sometimes makes someone extra vulnerable to other difficult feelings [ … ] when mothers are pregnant or have a baby, a lot of things from their own childhood come back [ … ] which makes it [a] very vulnerable [period] to use certain coping mechanisms from the past. And if that coping mechanism is resorting to drugs or alcohol or something like that, then that’s a difficult one too…”* (HC-2)

At the institutional level, additional constraints were mentioned. A key concern is the absence of a prenatal legal framework that would allow for earlier, prenatal, or more assertive interventions.

*“These are the mothers wandering the streets, who no longer have a fixed address, who sabotage or jeopardize every form of collaboration. You can’t reach them anymore, not by phone, and they disengage from all forms of support [ … ] And there’s nothing we can do about it. [ … ] Legally speaking, everyone is up against the wall … It all depends on whether or not the mother is willing to cooperate [ … ] These are very often children who end up being placed immediately after birth through an emergency protection order.”* (CPS-1)

*“I do think there are still a lot of opportunities in the future, but a clear framework from the government is really needed. And by framework, I mean legally—there really needs to be a way to intervene more assertively, even before the baby is born…”* (CPS-4)

Additionally, professionals raised concerns about the overemphasis on parental autonomy, which, although ethically grounded, may delay timely support and meaningful preparation for parenthood. Some questioned whether this approach can still be called adequate care:

*“We’re really standing with our feet in the mud; you can’t do anything prenatally. You can’t impose anything on parents, you have to walk on the delicate line of ‘If I’m too clear, I’ll lose them, they’ll flee, if I’m not clear enough, yeah, then it’s just [doing something]’, so you actually have to constantly think back and forth about how you’re going to say or achieve certain things.”* (HC-9)

*“It is kind of ambiguous, because you can say ‘Everyone has the right to do what they want with their life [ … ] ‘Okay, just go ahead, we can’t say anything anyway and … but maybe you’ll run into us after giving birth…’ Yeah, is that good care? Is that what we want? We must prepare people, eh medically but also psychosocially, for the role, the role, the role they are about to take on.”* (HC-9)

Not intervening was not only regarded as potentially harmful but also as ethically problematic, both for the (unborn) child and the mother. The lack of proactive engagement denies pregnant women with SUD the support they need to prepare for parenthood, undermining their opportunities for meaningful change. Rather than protecting autonomy, inaction may deepen marginalization and reinforce missed chances for recovery.

*That’s not right, that’s not right for the baby, but actually also not quite right for the mother, I think. Because yeah, you’re actually taking away her opportunity to be a good mother, by just … yeah, not giving yourself the power to do certain things.”* (HC-9)

### 3.2 Opportunities and facilitators to achieve change according to professionals

Professionals also identified several opportunities for change during the perinatal period. These included personal opportunities, such as the transformative role of motherhood as a source of motivation and identity shift, and contextual openings, particularly the increased and more consistent contact with healthcare services. In addition, professionals described several facilitators that could enhance the transformative potential of this period, including reproductive empowerment and embodied connection, the strengthening of professional capacity and relational practices, and the development of an integrated and responsive care system that more effectively supports pregnant and parenting women with SUD across sectors.

#### 3.2.1 Supporting reproductive empowerment and embodied connection

Professionals emphasized the need to engage in non-stigmatizing conversations about family planning with all women with SUD. First, psychoeducation, particularly to address misconceptions and promote informed choices around fertility and substance use, should be offered.

*“What I personally find important is that everyone feels like, ‘Okay, I understand that you have a desire to have children and that you want to be pregnant, ‘ so we actually try to approach that in a non-judgmental way. [ … ] Also, that, ‘[It’s] very normal, anyone can have a desire to have children, so why should you be any different…’”* (SU-4)

Further, discussions about appropriate contraception are essential. Long-acting reversible contraception (LARC) was often recommended, as SUD and related lifestyle factors may interfere with daily compliance:

*“Forget taking pills, that’s just not, not, not realistic”* (HC-10)

Professionals also underscored the importance of removing practical barriers, such as long waiting lists and costs. Some local projects have made contraception immediately accessible through direct collaborations.

*“We have the general practitioner across from us who inserts the implants. So, the moment someone, for example, says something about it, or says ‘I want contraception’, it can almost happen immediately. We also have a direct arrangement with [hospital] for the insertion of IUDs, which we pay for, which are placed there.”* (SU-10)

During pregnancy, professionals highlighted the importance of ongoing psychoeducation about the risks and consequences of substance use, even when the woman does not disclose current use. These conversations can lead to important turning points. Psychoeducation should also be offered by various professionals, including midwives, doctors, social workers, and mandated care staff. Each professional may offer a unique perspective, making the message more relatable and reinforcing its importance.

*“Like, it is unbelievable how little people sometimes consider the potential consequences of their substance abuse, and when you bring it up at the time of pregnancy [ … ]it does indeed open the eyes for some people [ … ].”* (HC-7)

However, since some parents with SUD may show some cognitive difficulties in retaining information, repetition and reinforcement by multiple professionals are essential to ensure understanding and retention.

*“Because people that [have substance use problems], who use [substances], are sometimes limited in being able to concentrate, so if you do an explanation of half an hour, sometimes you’ve already lost them after five or after ten minutes.”* (SU-11)

Secondly, professionals emphasized the need to help women connect cognitively and emotionally with their pregnancy and the unborn child by focusing on physical transformations and somatic experiences during pregnancy. Body-oriented and trauma-sensitive approaches, particularly for women with long histories of trauma and disconnection from their bodies, are possible treatment options. Educating women about early childhood development, particularly the first 1001 days, drawing on the Infant Mental Health (IMH) framework, may help to make the pregnancy more tangible and to foster early attachment by reinforcing that it begins in the womb:

*“I think that’s definitely [important], [asking] ‘What do you feel, what do you notice, what…?’ Not predicting everything, but getting to it together to … a very crucial period…”* (SU-12)

*“That’s also why the importance of bringing in that Infant [ … ] that Infant Mental Health framework … that baby, actually already from, from the moment they know it … to bring it in, to make it present…”* (CW-11)

Although the integration of this perspective in adult care, such as substance use services, is often lacking, it may help make motherhood feel more concrete and achievable, and the child less abstract, especially for women who struggle to envision a future beyond their substance use.

*“The partners with expertise in adult care, including addiction services, don’t have expertise in working with infants and Infant Mental Health and the attachment relationship [ … ] And conversely, we see that in the child and youth sector, uhm, they often do have experience with infants—though not guaranteed everywhere, but most networks do—but they say, ‘If adult issues are at the forefront, then it’s not for us’.”* (HC-11)

#### 3.2.2 Motherhood as a motivational and identity shift

Professionals emphasized that the perinatal period itself offers many opportunities for change through processes of identity transformation and growing maternal motivation, since pregnancy and motherhood touch on the existential meaning of these women’s lives. Opportunities include intrinsic desires to protect the unborn child’s health, the well-being of the child, changing intergenerational family scripts, and avoiding repeating past mistakes —*“I’m going to do things differently from what I went through”* (CPS-3) — as well as internal (e.g., guilt and shame) and external pressures (e.g., child protection involvement).

*“The mother was hugely frightened by the effects of her use on her baby [ … ] when you see how severely your baby suffers if it has severe withdrawal symptoms, yeah, that for her mother’s heart…”* (CW-8)

*“Mothers who look at their child and realize, ‘Yes, this is the reason why I do it, because if I screw up now, I’ll lose him’.”* (SU-8)

Moreover, this identity transformation also unfolds on the social level, as one professional explained:

*“Being a parent has a certain status, and then you move closer to, uhm, the mainstream group, uhm. And sometimes that gives you a bit of a … an incentive to do something with it.”* (SU-11)

In addition, professionals stressed the importance of bodily experiences during pregnancy, childbirth, and breastfeeding. Physical transformations, such as a growing belly or feeling fetal movements, can make the idea of motherhood more concrete, helping some women become more aware of their changing roles. For some, this awareness only fully emerges after childbirth.

*“Uhm, sometimes you also notice that during the last two months, three months of pregnancy, they [become] more and more [aware], because their belly is also clearly visible, and they feel everything much more.”* (CW-3)

*“During pregnancy, it’s sometimes still a bit too abstract. But I have actually seen people turn a corner after giving birth.”* (SU-11)

#### 3.2.3 Enhancing professional capacity and relational practice

Professionals emphasized that their competencies and knowledge must be further supported. In particular, there was a strong call for more education and training among healthcare professionals, many of whom lack a basic understanding of SUD in general and the specific complexities during the perinatal period. Significant variation in both knowledge and approach among healthcare providers, especially gynecologists, was also described. Additionally, the limited emphasis on the psychosocial aspect of pregnancy by gynecologists — “*They see the belly but not what surrounds it” (SU-11) —* often resulted in inadequate detection and referral.

*“The gynecologists here at the hospital [ … ] said to me, ‘No idea what I’m supposed to do with that info.’ [ … ] So, they were like, ‘Yeah, and then what?’ [ … ] You can really notice a difference there, at the university hospital, they do see it as valuable information, but those gynecologists actually admitted, during that meeting [ … ] that they themselves aren’t really up to speed on ‘What is the impact of which substance, during which phase of pregnancy?’”* (SU-5)

*“Sometimes you have to kind of, yeah, rely on the goodwill or the willingness of the gynecologists to see that [substance use] or to want to see that and to refer it.”* (CW-6)

Professionals suggested that midwives should play a more prominent role when provided they are given adequate time, training, and support. Midwives were seen as well-positioned to recognize early signs of vulnerability, yet many struggle to initiate conversations about substance use.

*“Gynecologists, let’s be honest, are mainly focused on the medical side [ … ] I wonder, where is the well-being, the psychological well-being? [ … ] There are exceptions, but actually, it’s something that’s ingrained in society, that when you’re pregnant, you go to the gynecologist. Actually, we should almost get to the point where that goes hand in hand with, also a pathway beforehand with a midwife [ … ] That she would then take on more of the psychological well-being part, so that it can be more of a tandem.”* (CPS-1)

*“That midwives’ network admitted, in all honesty, that they actually never ask their pregnant women whether there is any drug use. Yes, they find that a very difficult step to take.”* (SU-5)

Professionals advocated for a paradigm shift, moving away from reactive and punitive approaches toward a preventive, health-focused model of care, emphasizing empowerment, early intervention, and relational support.

*“We should focus more on not punishing or taking [the child] away, but rather on ‘Hey, we’re going to help you.’ Not immediately sitting down with a juvenile court and foster care involved but empowering them [ … ] Treating addiction more like an illness and investing more effort into that socially.”* (SU-8)

#### 3.2.4 Building an integrated and responsive care system

The role of contextual opportunities for change, particularly the increased and more consistent contact with healthcare services during the perinatal period, was also noted. Prenatal consultations ensure that women come into contact with healthcare services. These appointments offer valuable moments to engage women in care trajectories, especially when there is some level of insight or openness to change. Even women who typically exhibit hesitance or reluctance in seeking healthcare services eventually engage in prenatal care to monitor their pregnancy progress.

*“There are few occasions in people’s lives that you … see a woman as intensively and as regularly as during a pregnancy. You see them every month, at the end every three weeks, two weeks, weekly even, sometimes even more frequently if ultrasounds come in between, so you can basically engage in a process, a trajectory with that woman.”* (HC-9)

*“Also, people who initially previously avoided [ … ] contact with care [ … ] now have no choice but to … Yeah, they now actually do need to get in touch with care, for the sake of following up on that pregnancy and supervising childbirth.”* (HC-11)

Since multiple healthcare, child welfare, child protection services, and substance use services needs are intertwined, professionals stressed that addressing them requires intersectoral collaboration. Professionals emphasized that building bridges between sectors could facilitate earlier detection and more timely interventions. They emphasized the urgent need for more accessible and specialized care facilities tailored to the needs of pregnant and parenting women with SUD. Residential facilities that accommodate both mother and child remain scarce, and currently, no residential substance use settings exist that allow couples with SUD to reside together with their child. In addition, outpatient services with expertise at the intersection of pregnancy, parenting, and SUD are often locally organized, resulting in regional disparities and unequal access to appropriate care.

*“There are many people with a substance use problem who, I think, could be monitored or helped on an outpatient basis, and if that could be closer to the source, namely obstetrics, I think that could be a gain.”* (HC-3)

Some promising examples of integrated care were noted, such as embedding psychiatric support directly within obstetric services. This made psychosocial care more accessible and reduced stigma:

*“As a psychiatrist, I join the obstetrics department because people are pregnant, and then you notice that people are open to it, that there is openness”* (HC-3)

Nevertheless, such efforts often remain confined within hospital walls.

*“That’s a bit typical of the healthcare sector — it’s like, ‘We’ll just solve it all ourselves, we can handle everything’, and then I think, ‘No, people, you can’t do that, you’re not experts in all those things, you have to work together with experts, with support services, with child welfare, with guidance, with…’”* (HC-9)

Professionals also described the practical and structural challenges of implementing collaborative care that considers both mother and baby. One major issue is how to represent the baby’s perspective in adult care systems:

*“And if adult care is involved or needs to be involved: How can we then collaborate, who can then bring in the voice of the baby, well, how can we bring in the voice of the baby?”* (CPS-6).

Underlying these difficulties are structural barriers stemming from separate funding streams and responsibilities across adult and child welfare sectors:

*“The child and youth networks are funded differently than the, uhm, adult networks. And that means that when it comes to perinatality, we’re dealing with a division, there’s a barrier”* (HC-11).

Professionals further reflected on the recent policy discussions calling for compulsory treatment measures. However, many were skeptical about this evolution, highlighting the lack of adequate voluntary support services as a more pressing concern and voicing concerns about potentially counterproductive consequences:

*“A forced admission is quite drastic and a punishment, and actually, our basic care offer is still very limited, yes, underdeveloped for that group.”* (HC-11)

*“ldquo;Practice teaches us that, well, reacting that way doesn’t work. You will only create a subgroup that will hide them even more from care services, so that children will be born in squats and essentially kept hidden for a long time, so that they can’t be traced.”* (SU-6)

## 4 Discussion

This study explored how professionals working across healthcare, child welfare, substance use, and child protection services perceive the perinatal period as a window of opportunity for change among pregnant and parenting women with SUD. An important consideration when interpreting these findings is the complexity surrounding the definition of SUD in perinatal populations. While DSM-5 criteria provide a formal diagnostic framework, in practice, the distinction between substance use and SUD often becomes blurred during pregnancy and postpartum, partly due to (temporarily) changed SU patterns. Clinical and empirical assessments frequently conflate any substance use with SUD, influenced by contextual factors such as different professional opinions of what constitutes problematic use, perceived fetal or infant risk, and regional care norms (e.g., variation in treatment options and differences in providers’ knowledge and expertise regarding perinatal substance use). These differences introduce heterogeneity into the category of “women with SUD“, which may affect professionals’ perceptions of the perinatal period as a window of opportunity.

The findings highlight barriers to change at the level of pregnant and parenting women with SUD, professionals, and the broader care system, including disconnected bodily awareness, mutual avoidance between caregivers and women with SUD, limited insight and (self-) reflection on the impact of SUD and motherhood, and insufficient support networks and systemic gaps. However, the findings also point to several facilitators and opportunities, including reproductive empowerment and embodied connection, the transformative role of motherhood as a source of motivation and identity shift, enhanced professional capacity and relational practice, and the development of integrated and responsive care. The results confirm earlier research describing this period as a potential catalyst for change (22, 38), yet also emphasize its paradoxical nature: It is a time that may generate motivation and openness to care (54, 55), while simultaneously representing a period of intense vulnerability and the risk of punitive responses (36, 56).

Despite many barriers, professionals described naturally emerging opportunities within the perinatal period that can be recognized and leveraged for support, suggesting a gradual shift away from a solely deficit-based view (19, 49) toward a more nuanced, strengths-oriented understanding of perinatal SUD. Personal opportunities include the identity transformations associated with the transition to motherhood, such as the desire to meet internalized or societal expectations of “good motherhood“, to (be able to) care for the (unborn) child, and/or to break with the past. Moreover, professionals observed that becoming a mother can trigger reflection on women’s own childhoods and relationships with their mothers, activating intergenerational dynamics and, in some cases, a desire to “do better” through corrective scripts (57–59). These processes offer an opportunity to work through painful experiences and prevent their transmission to the next generation, while simultaneously reinforcing positive memories and internalized resources that can support the mothering role. Substance use services should therefore create space for such reflective processes within care trajectories. Additionally, and consistent with earlier studies (20, 22, 40), the increased and more sustained contact with healthcare professionals during pregnancy was seen as a contextual opportunity to initiate engagement and build supportive therapeutic relationships. Beyond these personal and contextual opportunities, professionals referred to several facilitators at the maternal, professional, and systemic levels that could enhance the transformative potential of the perinatal period.

At the same time, however, professionals across all four sectors included in this study emphasized that the extent to which opportunities and facilitators can be recognized and acted upon largely depends on the role of caregivers and the broader care system. Professionals across all four sectors acknowledged that caregivers and the broader care system not only play a crucial role in shaping whether and how the perinatal period can become a window of opportunity for change, but also in the emergence and persistence of barriers.

A recurring theme was reciprocal avoidance: It was described how caregivers sometimes deliberately avoided screening, detection, or referral out of discomfort, uncertainty, or fear of harming the therapeutic relationship. This mutual distancing contributes to missed opportunities for early identification and intervention. Participants pointed to a general lack of action around SUD in pregnancy, which cannot only be attributed to knowledge gaps but also to implicit stigma and attitudinal barriers (21). These findings align with previous literature highlighting structural and enacted stigma within healthcare settings (22–24), where SUD is often perceived as a personal choice or moral weakness (21). The results reinforce the importance of professional training and education, particularly in healthcare (60). A recent systematic review showed that stigma among perinatal healthcare professionals was present across all reviewed studies (61). As stigma can underlie poor interactions, reduced engagement, and treatment gaps, targeting training on substance use and perinatal care is essential (62). Evidence indicates that increased expertise in substance use correlates with more positive attitudes toward pregnant and parenting women with SUD (49, 63) and that sensitive, non-judgmental approaches foster engagement (64). Moreover, stigma not only shapes clinical interactions but also operates at the systemic level, influencing policy priorities and investment decisions. Underinvestment in substance use services and the limited availability of specialized care for pregnant and parenting women with SUD (21, 64) both reflect and perpetuate stigma, sustaining punitive rather than therapeutic responses, discouraging access to services, and creating a self-reinforcing cycle of marginalization (21, 64, 65).

Another recurring theme was the need to adopt a broader lens that includes preconception care. While previous research has rightly emphasized the importance of the so-called “fourth trimester” — the early postpartum period marked by increased vulnerability and the need for continued support (66) — professionals in this study extended this view by underscoring the relevance of the period before pregnancy. In addition to established harm-reduction strategies, such as needle exchange, HIV prevention, and methadone substitution, they called for a stronger focus on contraceptive care for all women of reproductive age with SUD (67, 68). Several professionals highlighted promising local projects offering free, long-acting, reversible contraception; however, these were not structurally embedded. Moreover, there should be more awareness among all professionals who encounter women with SUD, including general practitioners, social workers, and professionals within substance use services, to proactively discuss topics such as menstruation, reproductive health, and family planning. Such efforts should not only be centered on the woman but also the potential child, reinforcing the need for early, rights-based approaches to reproductive health, pregnancy planning, and prevention.

These findings, in line with previous research highlighting the value of integrated care (26, 27), underscore the need for intersectoral collaboration. Professionals described the current care system as fragmented, with insufficient integration between healthcare (e.g., general practitioners, midwives, gynecologists) and substance use services. Given the central role of healthcare professionals during pregnancy, ensuring adequate reproductive health and access to contraception for women with SUD requires close collaboration between sectors (69). As such, structurally embedding obstetric expertise within substance use services, improving access to obstetric care from within these services, and establishing clear pathways for low-threshold referral between medical and substance use services could significantly enhance integrated care, ensure continuity of care, and improve earlier and more effective interventions in the domain of reproductive health.

Moreover, participants problematized the division between adult services and child welfare, given that the perinatal period in particular necessitates close and continuous collaboration between both sectors. During the interviews, the Infant Mental Health (IMH) framework was highlighted, especially by professionals in child welfare, as a transformative model since it shifts the focus from mere substance use to the broader developmental and relational context of emerging motherhood, supporting early bonding and attachment and the emotional and psychological needs of both mother and (unborn) child (70). Moreover, from a motivational perspective, IMH may facilitate the internalization of extrinsic motivation, such as external pressure, by encouraging feelings of relatedness to the (unborn) child (71). Building on this, it can be argued that there is a clear need to evolve from a traditional IMH approach toward a more integrated Parent Infant Mental Health (PIMH) framework—one that is embedded across both adult (mental) health and child welfare systems, ensuring sustained attention to the needs of both the mother and the (unborn) child. In line with the “no wrong door” principle (72), appropriate support should be accessible in any care context, irrespective of where or how a woman engages with services. The point at which a pregnant woman enters the care system—or whether she enters it at all—may be a decisive factor in ensuring both her medical and psychosocial stability, as well as the long-term health and development of the child.

Consistent with previous research (69), a need for more emphasis on the psychosocial aspect of pregnancy within obstetric care was another recurring theme. Participants suggested giving midwives a more prominent role, provided they are given adequate time, training, and support. Simultaneously, the findings echo the need to incorporate body-oriented approaches into substance use services (73), particularly for women with trauma histories, as promoting the ability to gain bodily awareness is fundamental for behavior change and rebuilding the interoceptive process that is disrupted by SUD (74–77).

A final theme concerned the growing discrepancy between policy discourse and clinical practice regarding SUD during pregnancy. Professionals expressed concern about recent policy trends that lean toward coercion—such as debates around compulsory treatment—which were seen as potentially counterproductive. Professionals feared that such measures would reinforce stigma and further alienate women from seeking care. At the same time, professionals criticized the absence of a legal and policy framework for prenatal care and SUD. In a context where pregnancy shifts the boundaries between private life and public responsibility, shifting what was once a personal matter into a societal concern (40), professionals felt caught between ethical care and structural limitations. There was a shared call for a legal framework that enables early, supportive, and non-punitive intervention, aiming to both safeguard the unborn child and to empower the pregnant woman. This tension also raised complex ethical questions about how to balance maternal autonomy with fetal well-being (63). While professionals emphasized that coercion should remain a last resort, they primarily underlined the need for more specialized facilities that can offer accessible, timely, voluntary, and appropriate support.

These concerns reflect broader societal dynamics in which fetal protectionism increasingly shapes policy (56). In some countries, parental SUD is criminalized, further framing pregnant women with SUD as morally deviant or neglectful (43). These deficit-oriented discourses risk creating a self-fulfilling prophecy: stigma and fear deter women from seeking help, and those who do engage with services often encounter judgment, making it more difficult to meet the idealized expectations of motherhood (21, 23, 24). The mismatch between punitive policy orientations and non-punitive, evidence-based practice recommendations has been previously documented (62, 66), and was echoed by professionals in this study. Participants called for a realignment of policy frameworks with person-centered, relational, and strengths-based approaches.

Challenging deficit discourses is therefore essential, as these narratives not only obscure structural inequalities but also shape professional attitudes and interactions with vulnerable populations (78). A strengths-based approach is not simplistic, nor does it deny the reality of adversity; rather, it intentionally highlights individual capacities, contextual resources, and relational potential for change (72, 78). This entails a shift in how vulnerability is conceptualized: not as a marker of deficiency, but as a site of ethical engagement and opportunity, taking into account realistic expectations. As such, it is essential to move beyond hegemonic discourses that frame strength and deficit, mothering and substance use, or challenge and opportunity as binary opposites. Instead, we must recognize that these dimensions often coexist and intersect, and that acknowledging this complexity is crucial for developing responsive, non-reductionist, and ethically sound models of care.

### 4.1 Limitations and future research

This study is part of a PhD project examining the perinatal period as a window of opportunity for change among pregnant and parenting women with SUD. The first study of this research project provides a comprehensive, cross-sectoral exploration of perinatal care for women with SUD by including professionals from four key sectors: healthcare, substance use services, child welfare, and child protection. The selection of services enabled identification of both overarching themes and sector-specific nuances, reflecting the complexity of perinatal substance use care and support. Future studies will incorporate the voices of mothers themselves, allowing for a more direct understanding of their experiences, needs, and challenges within the perinatal trajectory.

Another important consideration for future research is the lack of a clear operational definition of perinatal SUD. In this population, the distinction between any substance use and a diagnosable disorder is often fluid and context-dependent. Future studies should acknowledge this complexity and make explicit how ‘substance use’ was operationalized, as clearer definitions can help to improve comparability across studies and guide clinical decision-making. Moreover, longitudinal studies that follow up women with varying levels of substance use—those meeting diagnostic criteria for a SUD and those who do not—can clarify whether the perceived “window of opportunity” for change differs between women with clinical (diagnosed) and sub-clinical patterns of substance use.

This study has several limitations that should be considered when interpreting the findings. First, although the data reflect the perspectives of professionals across multiple sectors, they do not capture the lived experiences of women with SUD. Consequently, the findings offer an indirect view of how opportunities, facilitators, and barriers for change in the perinatal period are perceived by professionals, rather than how they are experienced and navigated by women with SUD themselves. Future work will integrate the perspectives of mothers to complement these findings and provide a more comprehensive understanding of perinatal care and recovery pathways.

Second, the sampling strategy may have introduced a degree of selection bias. The study was initiated within specialized substance use services with particular expertise in supporting women with SUD and their children in the perinatal period. Recruitment then proceeded through snowball sampling, with the aim of including professionals with relevant expertise across the different sectors. While this approach allowed for the inclusion of information-rich participants, it may have resulted in an overrepresentation of professionals who are already sensitized to the topic and committed to providing supportive care. As a result, the findings may reflect more progressive responses than might be found in the broader professional field. Furthermore, due to data saturation, professionals from child protection services were underrepresented in the final sample, limiting the breadth of perspectives from that domain.

Finally, as in all research, there is a potential bias of social desirability and professional positioning. In this study, participants may have portrayed their own practices of sectoral norms in a more favorable light, particularly in relation to sensitive topics such as stigma, coercion, or perceived shortcomings with the broader care system. Such tendencies could have resulted in more idealized accounts of care practices and underreporting of challenges or ethical dilemmas. Nevertheless, their perspectives provide valuable insights into a typically marginalized field. To mitigate bias, a semi-structured interview guide was used, and coding and interpretation were discussed among multiple authors, enhancing reflexivity and minimizing the impact of individual assumptions. Moreover, we performed a COREQ evaluation for reporting the results (51).

## 5 Conclusion

This study highlights how professionals across healthcare, child welfare, substance use, and child protection services perceive the perinatal period as a critical window of both vulnerability and opportunity for women with SUD. Importantly, the findings point to a gradual shift away from deficit-focused narratives toward a more strengths-based understanding of perinatal substance use.

However, realizing the transformative potential of the perinatal period requires more than individual motivation or professional goodwill. It demands integrated, cross-sector care pathways that promote early engagement and sustained support, improved professional training, greater attention to body-oriented approaches, and more attention to reproductive and preconception care across all sectors. Such an approach acknowledges the perinatal period not simply as a time of risk or promise, but as a complex juncture shaped by personal, social, relational, and structural influences, requiring ethically grounded and responsive care models that support both the mother and (unborn) child.

## Data Availability

The raw data supporting the conclusions of this article will be made available by the authors, without undue reservation.
